# Mutational profiling of non-small-cell lung cancer patients resistant to first-generation EGFR tyrosine kinase inhibitors using next generation sequencing

**DOI:** 10.18632/oncotarget.11237

**Published:** 2016-08-12

**Authors:** Ying Jin, Yang Shao, Xun Shi, Guangyuan Lou, Yiping Zhang, Xue Wu, Xiaoling Tong, Xinmin Yu

**Affiliations:** ^1^ Department of Medical Oncology, Zhejiang Cancer Hospital, Hangzhou, China; ^2^ Zhejiang Key Laboratory of Radiation Oncology, Hangzhou, China; ^3^ Geneseeq Technology Inc., Toronto, Ontario, Canada; ^4^ Zhejiang Key Laboratory of Diagnosis and Treatment Technology of Thoracic Oncology, Hangzhou, China

**Keywords:** non-small-cell lung cancer, epithelial growth factor receptor, tyrosine kinase inhibitor, drug resistance, next generation sequencing

## Abstract

Patients with advanced non-small-cell lung cancer (NSCLC) harboring sensitive epithelial growth factor receptor (EGFR) mutations invariably develop acquired resistance to EGFR tyrosine kinase inhibitors (TKIs). Identification of actionable genetic alterations conferring drug-resistance can be helpful for guiding the subsequent treatment decision. One of the major resistant mechanisms is secondary *EGFR-T790M* mutation. Other mechanisms, such as *HER2* and *MET* amplifications, and *PIK3CA* mutations, were also reported. However, the mechanisms in the remaining patients are still unknown. In this study, we performed mutational profiling in a cohort of 83 NSCLC patients with TKI-sensitizing *EGFR* mutations at diagnosis and acquired resistance to three different first-generation EGFR TKIs using targeted next generation sequencing (NGS) of 416 cancer-related genes. In total, we identified 322 genetic alterations with a median of 3 mutations per patient. 61% of patients still exhibit TKI-sensitizing *EGFR* mutations, and 36% of patients acquired *EGFR-T790M*. Besides other known resistance mechanisms, we identified *TET2* mutations in 12% of patients. Interestingly, we also observed *SOX2* amplification in *EGFR-T790M* negative patients, which are restricted to Icotinib treatment resistance, a drug widely used in Chinese NSCLC patients. Our study uncovered mutational profiles of NSCLC patients with first-generation EGFR TKIs resistance with potential therapeutic implications.

## INTRODUCTION

Lung cancer is the leading cause of cancer death in China as well as worldwide [1]. Approximately 80% of lung cancers are non-small-cell lung carcinoma (NSCLC), and the overall 5-year relative survival rate for this cohort is less than 20% [2]. Patients with advanced NSCLC have an extremely high mortality rate. During the past decades, genomic research has increased our understanding of the molecular characterization of cancer [3–5]. The treatment strategy for advanced NSCLC has changed dramatically from the traditional chemotherapy depending on pathologic histology to personalized precision medicine based on the oncogenic drivers [6].

The epidermal growth factor receptor (*EGFR*) gene mutations are found in ~10% of lung adenocarcinomas in Caucasian population [3], but in 30% ~ 50% of Asian population [7, 8], which define a substantial population that can benefit from the use of EGFR tyrosine kinase inhibitors (TKIs). Several randomized phase III clinical trials have shown the superiority in the overall response rate (ORR) and median progression-free survival (PFS) of EGFR TKI treatment over chemotherapy as first-line therapy for patients with TKI-sensitizing *EGFR* mutations [9, 10]. However, the vast majority of patients inevitably experienced acquired resistance in less than one year, limiting the overall survival advantage of EGFR TKI treatment over chemotherapy [11, 12].

Currently, the known mechanisms of acquired resistance are as follows [13–17]: 1) the secondary gatekeeper *EGFR* T790M mutation which increases ATP affinity and subsequently prevents drug binding to the kinase domain; 2) activation of members of downstream signaling pathways such as RAS-RAF-ERK MAPK pathway and PI3K/AKT/mTOR pathway; 3) activation of bypass signaling through receptor tyrosine kinase such as MET; 4) changes in tumor histology with tumor cells displaying features of small-cell lung cancer or epithelial-mesenchymal transition (EMT). The above mechanisms account for about 70% of acquired resistance, with 30% of remaining patients demonstrating unknown resistant mechanisms.

The introduction of next generation sequencing (NGS) into cancer genetic interrogation achieved tremendous successes in acquiring cancer genomic information comprehensively and efficiently [18]. It demonstrates great potentials in identifying genetic aberrations that can be used to match targeted drugs and monitoring acquired genetic changes during the treatment with limit amount of tumor materials. To take advantage of this technology, we performed targeted NGS with a gene panel covering 416 cancer-related genes to profile genetic characteristics of 83 non-small cell lung cancer (NSCLC) patients after they developed systematically progress to the first generation EGFR TKI treatments, including erlotinib, gefitinib and icotinib. Besides *EGFR* T790M mutations, a variety of other previously known and novel genetic alterations were identified that might be potentially related to their primary and acquired resistance to treatments.

## RESULTS

### An overall characterization of cancer-related mutations identified in all patients

We analyzed either genomic DNAs from formalin-fixed paraffin embedded (FFPE) samples or pleural effusions, or circulating tumor DNAs (ctDNA) from plasma samples from 83 Chinese NSCLC patients with stage IV diseases at the time of developing drug resistance to the first generation of EGFR TKIs, erlotinib, gefitinib or icotinib. These patients were identified with TKI-sensitizing *EGFR* mutations prior to treatments and their characteristics were summarized in Table 1. The choice of collecting different tumor materials depends on clinical risks that would impose on the patients by the operation. 45 patients (54.2%) patients were undertaken blood withdrawing for testing ctDNA, while in others tumor tissues or pleural effusions were obtained through biopsies. Prior-treatment histology analysis confirmed that 68 patients (81.9%) were adenocarcinoma and 4 (4.8%) were squamous cell carcinoma. The rest 11 patients cannot be clearly distinguished based on histology appearance. Half of patients were subjected to icotinib treatment upon diagnosis largely because of its lower cost compared to the other two options [19].

**Table 1:** Patients' characteristics

characteristic	
Sex, No. (%)	
Female	36 (43.3)
Male	47 (56.6)
Age, years	
Median	61
Range	29~85
Histology, No. (%)	
Adenocarcinoma	68 (81.9)
Squamous cell carcinoma	4 (4.8)
unknown	11 (13.3)
Sample type, No. (%)	
FFPE	26 (31.3)
Plasma	45 (54.2)
Pleural effusions	12 (14.5)
EGFR-TKI history, No. (%)	
Gefitinib	26 (31.3)
Icotinib	42 (50.6)
Erlotinib	15 (18.1)

**Figure 1:**
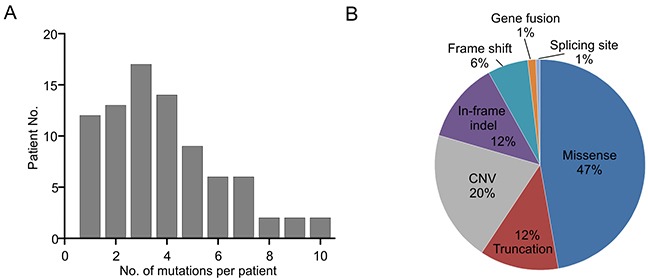
Mutation types and mutation number identified in 83 patients **A.** The number of mutations identified in each patient was plotted to a histogram. **B.** Total mutations detected in 83 patients were classified according to the mutation types.

A total of 322 cancer-related genetic mutations were detected in these patients with a median of 3 mutations per patient and a range of 1-10 mutations per patient (Figure 1A). 87 genes within the 416-gene panel were involved. Majority of mutations (47%) were missense mutations and other types of mutations were also uncovered (Figure 1B).

### *EGFR* mutational status in all patients

**Figure 2:**
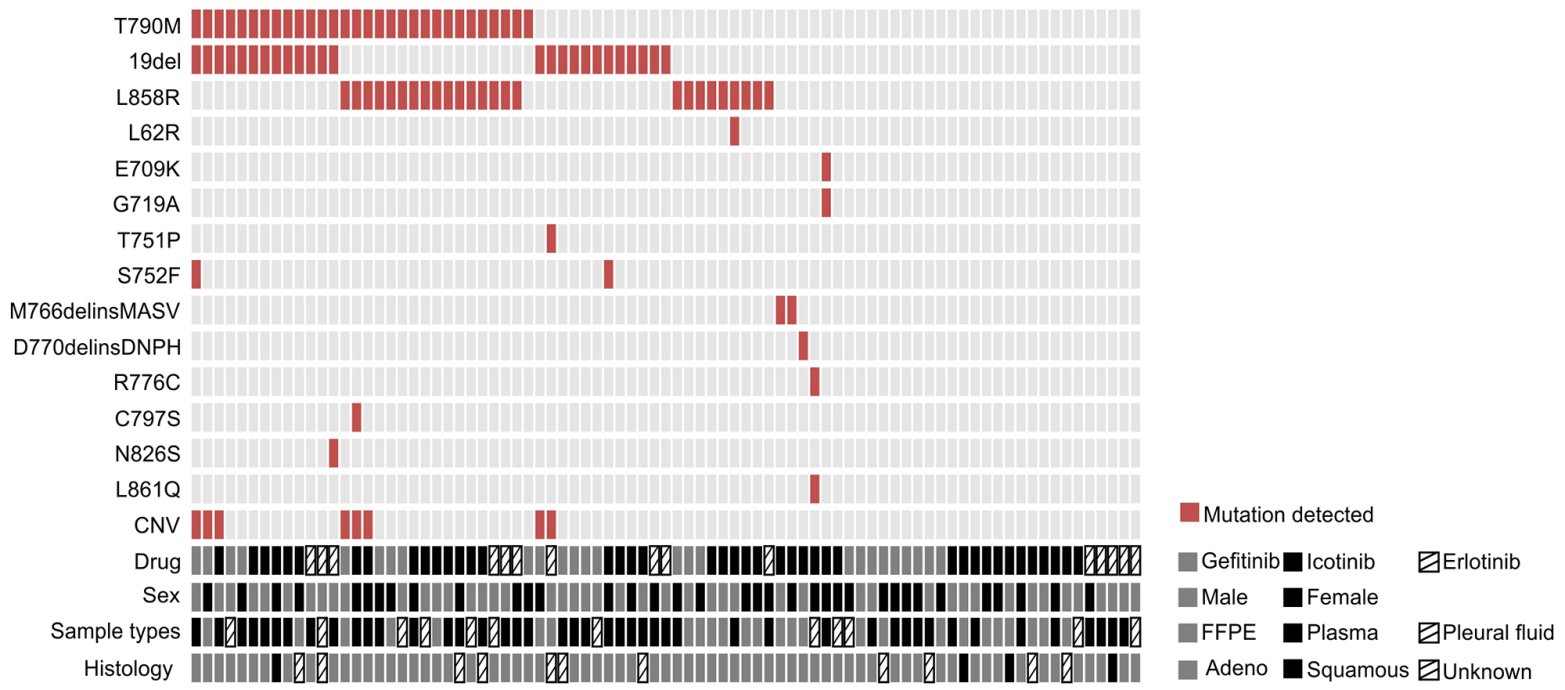
Comutation plot of EGFR mutations in 83 patients Each vertical line of blocks represents a patient. Patient features, including the drug they used, their sexes, tumor sample types that collected and histology types, were aligned below the mutation plot.

30 of 83 patients (36.1%) were detected with *EGFR* T790M mutation and all of them except one were found harboring *EGFR* activating mutation either exon 19 deletion (19del) or L858R (Figure 2). 6 of them were accompanied with the copy number gain of *EGFR* and one of them harbors C797S mutation, which will exert resistance to the third generation EGFR TKI, AZD9291 [20]. Uncommon mutations including S752F and N826S were also identified in one case each, which might be related to the resistance to gefitinib and erlotinib according to previous reports [21, 22].

As to the other *EGFR-T790M* negative (T790M-) patients, in addition to the presence of 19del (23%) and L858R (17%), a variety of other infrequent *EGFR* mutations that were suggested less sensitive to the first generation TKIs were identified, including M766delinsMASV, D770delinsDNPH, L861Q and G719A [23, 24], as well as R776C mutation that was previously reported to be more sensitive to erlotinib than gefitinib [21]. One patient carries doublet mutations E709K/G719A, which were reported as oncogenic drivers but maintaining the same responses to the first generation TKIs [25, 26]. 27 patients were not identified with any *EGFR* mutations. Statistical analysis indicates that the presence of T790M did not show clear association with gender or targeted drugs adminstrated (Table 2).

**Table 2:** Comparison of the most frequently mutated genes among patients with different characteristics

Mutated Genes	*EGFR* T790M No. (%)	*TET2* No. (%)	*SOX2* No. (%)	*MET* No. (%)	*KRAS* No. (%)	*ALK* No. (%)
Sex						
Female	12 (33.3)	4 (11.1)	1 (2.8)	2 (5.6)	1 (2.8)	0 (0)
Male	18 (38.3)	6 (12.8)	4 (8.5)	2 (4.3)	3 (6.4)	3 (6.4)
P value	0.818	1.000	0.382	1.000	0.629	0.254
**EGFR-TKI History**						
Gefitinib	9 (34.6)	4 (15.4)	0 (0)	4 (15.4)	1 (3.8)	0 (0)
Icotinib	15 (35.7)	6 (14.3)	5 (11.9)	0 (0)	2 (4.8)	1 (2.4)
Erlotinib	6 (40.0)	0 (0)	0 (0)	0 (0)	1 (6.7)	2 (13.3)
P value	0.910	0.358	0.109	0.015*	1.000	0.111

“%” indicates the percentage of each specific mutation detected in the population of defined category. The statistical differences of mutation frequency in different groups were tested by Fisher's exact test.

### Top mutated genes in T790M- patients

**Figure 3:**
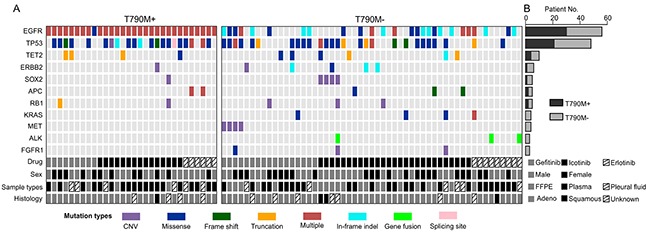
T790M+ and T790M- groups demonstrated different mutation spectrums **A.** Top 11 mutated genes (with at least 3 mutations identified in 83 patients) in T790M- group were selected and plotted against the T790+ group in order to compare the occurrence of different mutations between these two groups. Each vertical line of blocks represents a patient with patient features list at the bottom. Mutation types were differentiated by block colors. Multiple mutation types (red blocks) indicate that the patient have more than one mutations on the same gene. **B.** The number of mutated patients in T790M+ and T790M- groups was stacked for each gene. Each bar represents the gene on the left.

**Figure 4:**
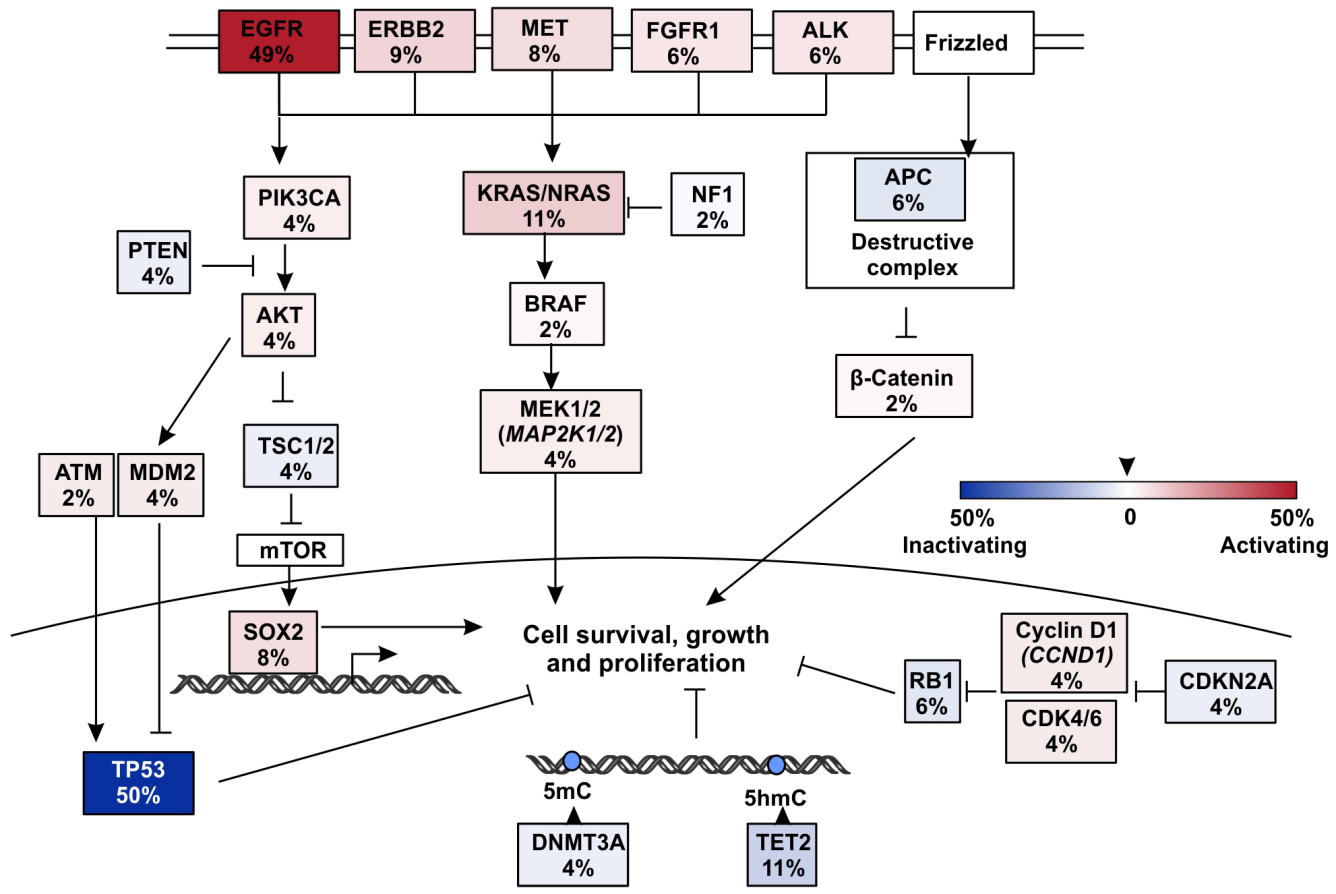
Pathways that were influenced by mutations in EGFR TKI resistant but T790M- patients Somatic mutations in all 53 T790M- patients were summarized and only key mutated genes were listed.

In T790M absent group, other genetic mutations are potentially responsible for drug resistance. To clarify this, we generated a co-mutation plot by dividing the patients into two groups according to their T790M statuses for 11 most frequently mutated genes in T790M- patients. T790M positive (T790M+) group showed limit number of mutations in these genes other than *EGFR* and *TP53* mutations compared to T790M- group, indicating that T790M alone is the dominant resistant mechanism in this group (Figure 3). In T790M- group, *EGFR* (26 out of 53 patients, 49%) and *TP53* (27, 50%) are still the most frequently mutated genes (Figure 3 and Figure 4). Other top mutated genes can be classified into following categories: 1) the activation and amplification of receptor tyrosine kinases (RTKs), including *ERBB2*, *MET* and *FGFR1*, and the rearrangement of *ALK* gene with *EML4*, *HERC1* and *HIP1*, respectively; 2) the activation of members of downstream RAS-RAF-ERK MAPK pathway, mainly including *KRAS*, *NRAS*, *MAP2K1/2*; 3) the activation of PI3K-AKT/mTOR pathway, correlated to the activated mutations of *PIK3CA*, *AKT*, *TSC1/2* and amplification of *SOX2* the downstream transcriptional factor; 4) disruption of epigenetic regulators, most noticeable *TET2* and *DNMT3A*; 5) the inactivation or copy number loss of tumor suppressor genes, including *APC*, *RB1* and *PTEN* (Figure 4 and Supplementary Table S1).

It is noticeable that *MET* amplification only occurred in gefitinib treated group and statistical analysis found a significantly difference of its occurrence in three treatment groups (*p* < 0.05) (Table 2). Meanwhile, *SOX2* amplification specifically presented in icotinib group and more frequently in male, but is not statistically different probably due to the small sample size (Table 2). The occurrence of *KRAS* activating point mutations is reported being mutually exclusive to *EGFR* mutations in treatment naïve patients possible due to their overlapping pathways in the onset of cancer [27, 28]. However, we observed one case that carries not only *KRAS* G13C and V14I, but also *EGFR* 19del, T751P and amplification (Figure 3A, Supplementary Table S1). It is also showed that the rearrangement of *ALK* only happens in cases without *EGFR* mutations, indicating its possible role in EGFR TKIs resistance.

We next determined the association of most commonly mutated genes with ages of patients at the time of NGS testing. Patients with *TP53*, *TET2* and *SOX2* mutations are all significantly older than those without mutations on these genes (Supplementary Figure S1). In contrast, the presence of *EGFR* T790M is not correlated with ages.

## DISCUSSION

This study profiled genetic backgrounds of 83 patients with advanced NSCLC using a targeted pan-cancer NGS panel after they developed resistance to three different first generation EGFR TKIs. To our knowledge, it is one of the largest panels in the clinical practice that covers most up-to-date cancer-related genes. Similar to previous reports, we identified *EGFR* T790M as the most abundant secondary mutation in developing drug resistance to the treatment of erlotinib, gefitinib and icotinib, and it is observed irrespective of sex, age and treatment options. The study depicts other diverse mechanisms that potentially are responsible for EGFR TKI resistance. The most frequent mechanisms in addition to *EGFR* T790M are the activation of other RTKs, including *ERBB2*, *MET*, *FGFR1* and *ALK* (totally 29% in T790M- patients), RAS/MEK/ERK pathway (totally 17%), as well as PIK3CA/AKT/mTOR pathway (totally 20%). RTKs are currently the most druggable targets. The representative drugs in market or in clinical trials are osimertinib (AZD9291) for *EGFR* T790M mutation [29], afatinib for *ERBB2* amplification, crizotinib for *ALK* gene fusions and *MET* amplification, and AZD4547 for *FGFR1* mutations.

Due to the lack of comprehensive genetic information before treatment, it is hard to conclude newly acquired resistant mechanisms solely from post-treatment results. But a few putative candidates that correlate to the resistance of the first generation EGFR TKI come to surface by comparing the genetic spectrums between *EGFR* T790M+ and T790- groups. *EGFR* T790M+ group did not encompass any mutations on genes *KRAS*, *MET*, *ALK* and *FGFR1* (Figure 3A), while all these genes have been suggested resulting in poor responses to EGFR TKIs in NSCLC patients [30–32]. Because both *MET* and *EGFR* reside on chromosome 7, a previous study suggested that polysomy of entire chromosome would result in the *in cis* amplification of *MET* and *EGFR* genes and it is hard to distinguish them by FISH [33]. Here in our study, 3 out of 4 cases with *MET* amplification were not escorted with *EGFR* amplification, and 1 case shows significantly higher amplification of *MET* compared to *EGFR*. Therefore, *MET* is more likely acting as a bypass signaling pathway that exerts resistance to EGFR TKIs. Moreover, it shows statistically higher occurrence in gefitinib-treated group rather than erlotinib and icotinib groups. Further studies with larger sample sizes might be needed in order to elucidate this finding.

Icotinib is the second line first generation EGFR TKI that was approved by China Food and Drug Administration (CFDA) in 2011, and because of its compatible efficacy and side effects as gefitinib [34], as well as its lower price, it is widely used in treating NSCLC in China. As far as we know, our study was the first to examine the genetic spectrums of icotinib-resistant NSCLC patients compared to other first generation EGFR TKIs, and found that *SOX2* amplification were private to icotinib treatment resistance. *SOX2* is frequently amplified at a percentage of around 20% in small cell lung cancer (SCLC) and NSCLC [35, 36]. It functions as a transcription factor that inhibits cell differentiation [37] and promotes cell cycling [38]. In addition, it is recognized as a downstream molecule of AKT/mTOR pathway that exerts controlling over cell survival [39].

There are several limitations in this study: firstly, since this was a retrospective analysis and most of patients were outpatients, we were unable to collect all the patients' subsequent treatment strategies and outcomes; Secondly, since there was no comparison with the pre-treatment genetic background, it was not certain whether these alterations have emerged as a result of acquired resistance, or they have been there since the beginning of the treatment; Thirdly, the relatively limited sample size of this study could decrease the statistical power in analysis. Thus, we are ready to conduct a prospective research using NGS technology to compare the pre-treatment and acquired resistance genetic profiles to further uncover the mechanism of resistance to EGFR-TKIs.

In summary, the study depicted the genetic landscapes comprehensively in Chinese NSCLC population resistant to first generation TKI treatments including icotinib. Our analysis demonstrates new perspectives for further study of resistance and putting forward corresponding relevant tactics against the challenge of disease progression.

## MATERIALS AND METHODS

### Patient enrollment and sample preparation

Between Jan 2015 to Dec 2015, a total of 83 patients with stage IV NSCLC in Zhejiang Cancer Hospital, China, were undergoing tumor biopsies or blood withdrawing by the time of acquiring resistance to the first generation EGFR TKIs, including 26 formalin-fixed paraffin-embedded (FFPE) samples, 45 serum samples and 12 serous effusions. Acquired resistance to EGFR TKIs was evaluated by “Jackman criteria” in each patient [40]. For FFPE samples, only samples harbored tumor cell content above 20% were considered qualified and included. Written consents from all patients were collected according to the ethic regulations of Zhejiang Cancer Hospital. Collected samples were sent to the core facility of Nanjing Shihe Jiyin Biotechnology Inc. (Nanjing, China) for targeted NGS analysis.

### DNA extraction

5-8 of 10μm tissue sections from tumor FFPE samples were used for genomic DNA extraction with QIAamp DNA FFPE Tissue Kit (QIAGEN) following the manufacturer's instructions. Plasma was extracted from 5-10 ml peripheral blood in EDTA-coated tubes within 2 hours of blood withdrawing, and circulating cell free DNA (cfDNA) was extracted using the QIAamp Circulating Nucleic Acid Kit (QIAGEN). Genomic DNA of cellular sediments of pleural effusions and whole blood samples were prepared with DNeasy Blood & Tissue kit (QIAGEN). Whole blood DNA was sequenced together with tumor or ctDNA samples for the purpose of identifying germline mutations. The DNA quality was assessed by Nanodrop2000 (Thermo Fisher Scientific) and the quantity was measured by dsDNA HS Assay Kit (Life Technologies) on Qubit 2.0.

### Library preparation and sequencing

Extracted tumor genomic DNA was fragmented into 300~350bp using Covaris M220 instrument (Covaris). Sequencing libraries were prepared with KAPA Hyper Prep kit (KAPA Biosystems) with optimized protocols. In brief, cfDNA or sheared tissue DNA were experienced with end-repairing, A-tailing, adapter ligation and size selection using Agencourt AMPure XP beads (Beckman Coulter). Libraries were then subjected to PCR amplification and purification before targeted enrichment.

DNA libraries from different samples were marked with unique indices during library preparation and up to 2 μg of different libraries were pooled together for targeted enrichment. Human cot-1 DNA (Life Technologies) and xGen Universal blocking oligos (Integrated DNA Technologies) were added to block nonspecific binding of library DNA to targeted probes. Customized xGen lockdown probes panel (Integrated DNA Technologies) were used to targeted enrich for 416 predefined genes. The hybridization reaction was performed by using NimbleGen SeqCap EZ Hybridization and Wash Kit (Roche). Dynabeads M-270 (Life Technologies) was used to capture probe-bind fragments, followed by library amplification with Illumina p5 (5′ AAT GAT ACG GCG ACC ACC GA 3′) and p7 primers (5′ CAA GCA GAA GAC GGC ATA CGA GAT 3′) in KAPA HiFi HotStart ReadyMix (KAPA Biosystems), and purification by Agencourt AMPure XP beads. Library quantification was analyzed by KAPA Library Quantification kit (KAPA Biosystems). The size distribution of libraries was measured by Agilent Technologies 2100 Bioanalyzer (Agilent Technologies). The enriched libraries were sequenced on Hiseq 4000 NGS platforms (Illumina) to coverage depths of at least 100x, 300x, 3000x after removing PCR duplicates for blood, FFPE/pleural effusion, and ctDNA, respectively.

### Annotation and interpretation of sequencing results

Trimmomatic [41] was used for sequencing data quality control. The reads with a quality below the threshold of 15, as well as N bases were removed before mapping to reference sequence hg19 (Human Genome version 19) using Burrows-Wheeler Aligner (BWA) [42] with optimized parameters. Genome Analysis Toolkit (GATK) [43] was used for indels local realignment and base quality score recalibration. SNPs/indels were identified using VarScan2 (MAF<10%) (http://dkoboldt.github.io/varscan/) and HaplotypeCaller/UnifiedGenotyper in GATK (MAF>10%). SNPs were filtered out with dbSNP and 1000 Genome data sets. Germline mutations in tumor tissues or ctDNA were identified by comparing to the matched whole blood DNA. A mutation was called when at least 3 mutated reads were found in the sample on different strands with good quality scores and manually inspected in Integrative Genomics Viewer (IGV, Broad Institute). Genomic fusions were identified by FACTERA [44] with default parameters. Copy number variations (CNVs) were detected using ADTEx (http://adtex.sourceforge.net) with default parameters. Proposed discrete wavelet transform (DWT) was used to reduce intrinsic noise.

## SUPPLEMENTARY FIGURE AND TABLE




