# Supplementary material for: Mutational profiling of non-small-cell lung cancer patients resistant to first-generation EGFR tyrosine kinase inhibitors using next generation sequencing
Source: Oncotarget. 2016 Aug 12;7(38):61755–63. doi: 10.18632/oncotarget.11237 (PMC5308688; doi:10.18632/oncotarget.11237)
Supplement: Supplementary file 1 [file oncotarget-07-61755-s001.pdf]

Mutational profiling of non-small-cell lung cancer patients resistant to first-generation EGFR tyrosine kinase inhibitors using next generation sequencing

SUPPLEMENTARY FIGURE AND TABLE

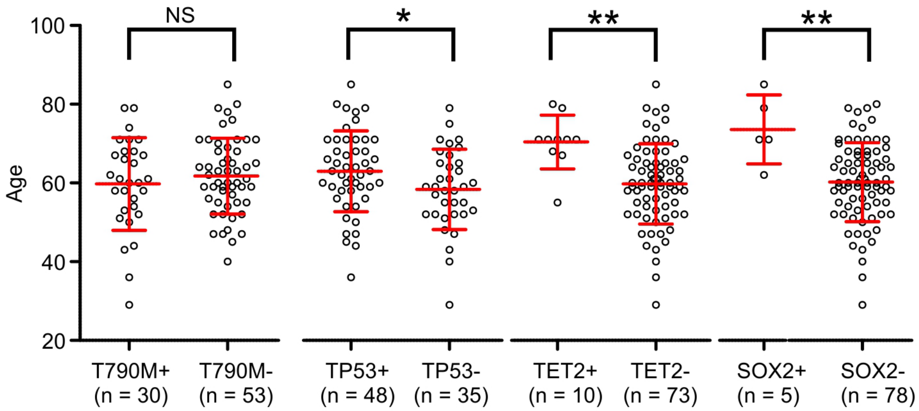

Supplementary Figure S1: Ages of patients with or without EGFR T790M, TP53, TET2 and SOX2 mutations in T790+ and T790- groups were statistically analyzed by Fisher's exact test. Each dot represents one patient and the error bar (red) indicates the mean and one standard deviation. * $p < 0.05$, ** $p < 0.01$.

Supplementary Table S1:

See Supplementary File S1
