# Supplementary material for: Mutational profiling of non-small-cell lung cancer patients resistant to first-generation EGFR tyrosine kinase inhibitors using next generation sequencing
Source: Oncotarget. 2016 Aug 12;7(38):61755–63. doi: 10.18632/oncotarget.11237 (PMC5308688; doi:10.18632/oncotarget.11237)
Supplement: Supplementary file 2 [file oncotarget-07-61755-s002.docx]

| Patient ID. | Gene Name | Mutation |
| --- | --- | --- |
| 003 | *EGFR* | L858R |
|  | *TP53* | E271X |
| 004 | *EGFR* | CNV |
|  | *EGFR* | 746_750del |
|  | *EGFR* | T751P |
|  | *KRAS* | G13C |
|  | *KRAS* | V141 |
|  | *MAP2K2* | CNV |
|  | *SMARCB1* | R374Q |
|  | *TP53* | E339X |
| 006 | *EGFR* | L858R |
|  | *EGFR* | T790M |
|  | *TP53* | D259A |
| 007 | *EGFR* | L858R |
|  | *EGFR* | T790M |
|  | *PIK3CA* | E545K |
|  | *ARID1A* | S2264X |
|  | *NF1* | G2527V |
| 008 | *STAG2* | K48R |
|  | *TET2* | E433X |
| 009 | *EGFR* | L858R |
|  | *EGFR* | T790M |
|  | *PIK3CA* | E545K |
|  | *APC* | P2669L |
|  | *APC* | E1464K |
|  | *APC* | S2575F |
|  | *TP53* | P72R |
|  | *TP53* | P151R |
| 010 | *NRAS* | G12S |
|  | *PRDM1* | S142X |
|  | *SMARCA4* | Q915X |
| 011 | *EGFR* | CNV |
|  | *EGFR* | T790M |
|  | *EGFR* | S752F |
|  | *EGFR* | 746_752del |
| 012 | *EGFR* | 746_750del |
|  | *CDKN1C* | 171_175del |
|  | *CDKN1C* | 183_187del |
| 013 | *EGFR* | L858R |
|  | *EGFR* | T790M |
|  | *TP53* | R174W |
| 014 | *ERBB2* | CNV |
|  | *AXL* | CNV |
|  | *ERBB4* | R95C |
| 017 | *EGFR* | CNV |
|  | *EGFR* | T790M |
|  | *EGFR* | 746_751del |
|  | *TP53* | R283P |
| 019 | *EGFR* | L858R |
|  | *EGFR* | T790M |
|  | *RB1* | Q575X |
|  | *RECQL4* | P1170L |
|  | *TP53* | P278R |
| 020 | *EGFR* | 746_751del |
|  | *AKT2* | CNV |
|  | *DNMT3A* | R409X |
|  | *NF1* | V2436L |
|  | *TP53* | Y220C |
| 021 | *EGFR* | L858R |
|  | *EGFR* | T790M |
|  | *DICER1* | I932T |
|  | *IDH1* | Q387X |
|  | *TET2* | Q1542X |
|  | *TP53* | G154fs |
| 025 | *ARID2* | K235fs |
|  | *GATA6* | R438L |
|  | *PRKCI* | CNV |
|  | *SDHA* | X665C |
|  | *SOX2* | CNV |
|  | *TET2* | S1898F |
|  | *TP53* | E271X |
|  | *TP53* | Y220C |
|  | *TP53* | CNV |
| 026 | *FAT1* | H3912fs |
|  | *NOTCH1* | P2220fs |
|  | *STK11* | K235X |
|  | *TP53* | E204X |
| 032 | *FLT4* | CNV |
|  | *AXL* | CNV |
| 034 | *ALK* | EML4-ALK |
|  | *AURKA* | A61T |
|  | *GNAS* | R201H |
| 035 | *STAG2* | Q1165X |
|  | *STAG2* | Q1168R |
| 040 | *EGFR* | L858R |
|  | *EGFR* | T790M |
|  | *ASXL1* | S689X |
|  | *TET2* | R1452X |
|  | *TP53* | E285K |
| 041 | *APC* | W50L |
| 044 | *EGFR* | CNV |
|  | *EGFR* | L858R |
|  | *EGFR* | T790M |
|  | *DNMT3A* | W313X |
|  | *EGFR* | C797S |
|  | *TP53* | CNV |
| 047 | *TP53* | R175H |
| 054 | *EGFR* | 746_750del |
|  | *EGFR* | T790M |
| 055 | *EGFR* | L858R |
|  | *PTEN* | 802-15_804del |
|  | *TP53* | C135F |
| 056 | *EGFR* | L858R |
|  | *EGFR* | T790M |
|  | *CCNE1* | CNV |
|  | *SDHD* | W130X |
|  | *TP53* | 177_182del |
| 059 | *BRCA1* | C903X |
|  | *ERBB2* | E770delinsEAYVM |
|  | *TP53* | E339X |
| 061 | *EGFR* | S752F |
|  | *EGFR* | 746_752del |
|  | *RUNX1* | R139Q |
|  | *TP53* | G266V |
|  | *TP53* | K320N |
|  | *TSC1* | S820X |
|  | *TSC1* | Q109X |
| 062 | *ERBB2* | E770delinsEAYVM |
|  | *MDM2* | CNV |
| 063 | *CCND1* | CNV |
|  | *MCL1* | CNV |
|  | *PTEN* | E40X |
|  | *RECQL4* | Q800X |
|  | *SOX2* | CNV |
|  | *TP53* | V274A |
| 065 | *EGFR* | M766delinsMASV |
|  | *FLT4* | CNV |
|  | *PIK3CA* | H1047R |
|  | *CCNE1* | CNV |
|  | *MYCN* | CNV |
|  | *NOTCH1* | CNV |
|  | *RB1* | CNV |
| 066 | *EGFR* | L858R |
|  | *EGFR* | T790M |
|  | *FLT4* | CNV |
|  | *CEBPA* | E92X |
|  | *DNMT3A* | R598X |
|  | *TET2* | Q847X |
| 069 | *EGFR* | 746_751del |
|  | *EGFR* | T790M |
|  | *DNMT3A* | V502fs |
|  | *TP53* | V216L |
| 072 | *SETD2* | R1496X |
| 075 | *EGFR* | L858R |
|  | *ARID2* | Q1462X |
|  | *EGFR* | L62R |
|  | *HNF1A* | G288fs |
|  | *TP53* | L252fs |
| 079 | *EGFR* | 746_750del |
|  | *EGFR* | T790M |
|  | *CDK4* | CNV |
|  | *CTNNB1* | S45P |
|  | *CTNNB1* | 33_36del |
|  | *KDM5A* | CNV |
|  | *MDM2* | CNV |
| 080 | *CDK8* | A172S |
|  | *ERBB2* | L846F |
|  | *MDM2* | CNV |
|  | *SOX2* | CNV |
|  | *TP53* | L194R |
| 082 | *ATM* | E708G |
|  | *CDKN2A* | V115E |
|  | *HDAC2* | E63X |
|  | *MEN1* | L301fs |
| 084 | *KRAS* | G12V |
|  | *CDKN2A* | P81R |
|  | *GRM3* | R183H |
|  | *TP53* | V122fs |
| 086 | *EGFR* | L858R |
| 087 | *EGFR* | 746_750del |
| 088 | *EGFR* | L858R |
|  | *EGFR* | T790M |
|  | *POLE* | 1088_1092del |
|  | *TP53* | I195F |
| 090 | *EGFR* | L858R |
|  | *TP53* | H179Q |
| 091 | *EGFR* | 746_750del |
|  | *TP53* | R175G |
| 096 | *EGFR* | CNV |
|  | *EGFR* | L858R |
|  | *EGFR* | T790M |
|  | *TP53* | 212_215del |
| 097 | *EGFR* | M766delinsMASV |
|  | *TP53* | P177R |
| 098 | *EGFR* | L858R |
|  | *APC* | N1984fs |
|  | *TP53* | R273L |
| 103 | *EGFR* | L858R |
|  | *CSF1R* | G765D |
|  | *RB1* | CNV |
| 106 | *EGFR* | 746_750del |
|  | *EGFR* | T790M |
|  | *APC* | E221fs |
|  | *APC* | K222X |
|  | *CBL* | R420X |
|  | *CDKN2A* | P41fs |
|  | *PTEN* | 197_198del |
|  | *TP53* | A560-2G |
|  | *TP53* | CNV |
| 108 | *EGFR* | 746_750del |
|  | *EGFR* | T790M |
|  | *EGFR* | CNV |
| 112 | *ALK* | HERC1-ALK |
|  | *FGFR1* | CNV |
|  | *TP53* | R175H |
|  | *CCND1* | CNV |
|  | *MYC* | CNV |
|  | *RB1* | CNV |
|  | *SOX2* | CNV |
| 114 | *FLCN* | K462fs |
|  | *KDR* | N402K |
|  | *RECQL4* | E796X |
|  | *SETD2* | V990fs |
| 116 | *TET2* | R1167K |
| 118 | *EGFR* | 746_750del |
| 120 | *EGFR* | 747_753del |
|  | *EGFR* | T790M |
| 121 | *EGFR* | 745_750del |
| 123 | *EGFR* | L858R |
|  | *EGFR* | T790M |
|  | *PTPN11* | A72V |
|  | *TP53* | S240R |
| 125 | *EGFR* | 745_750del |
|  | *MET* | CNV |
|  | *TP53* | I195T |
|  | *JAK2* | S507X |
| 127 | *EGFR* | 745_750del |
|  | *EGFR* | T790M |
|  | *TP53* | R248L |
| 128 | *EGFR* | L858R |
|  | *EGFR* | T790M |
|  | *EGFR* | CNV |
|  | *TP53* | C135Y |
|  | *IL7R* | CNV |
|  | *RICTOR* | CNV |
| 129 | *ABL1* | BCR-ABL1 |
|  | *DNMT3A* | L738Q |
|  | *NRAS* | G12D |
| 132 | *EGFR* | 746_750del |
|  | *EGFR* | T790M |
|  | *EGFR* | N826S |
|  | *PIK3CA* | H1047R |
| 133 | *ERBB2* | E770delinsEAYVM |
|  | *TET2* | R1167K |
|  | *TP53* | L257V |
| 136 | *EGFR* | L858R |
|  | *MET* | CNV |
|  | *TP53* | Y163C |
| 137 | *ASXL1* | Q882X |
|  | *ASXL1* | E929X |
|  | *CDK4* | CNV |
|  | *CDK6* | CNV |
|  | *FGFR1* | CNV |
|  | *KRAS* | G12V |
|  | *MSH2* | 1904fs |
|  | *MYC* | CNV |
|  | *TET2* | Y1766_S1767delinsX |
|  | *TP53* | C242F |
| 139 | *KRAS* | G12C |
|  | *TP53* | I162S |
| 141 | *EGFR* | 746_750del |
|  | *EGFR* | T790M |
|  | *ERBB2* | CNV |
|  | *TP53* | R342fs |
|  | *CCND1* | CNV |
|  | *FGF19* | CNV |
| 143 | *EGFR* | D770delinsDNPH |
| 145 | *EGFR* | T790M |
| 148 | *EGFR* | L858R |
|  | *MET* | CNV |
|  | *TP53* | S94X |
|  | *TP53* | Q104X |
|  | *TP53* | R110C |
|  | *FGFR1* | E138K |
|  | *KMT2A* | H2371Y |
| 150 | *CTNNB1* | S37F |
|  | *TET2* | Q591X |
| 151 | *EGFR* | 746_750del |
|  | *EGFR* | T790M |
|  | *PIK3CA* | E542K |
|  | *TP53* | V216L |
| 152 | *EGFR* | 746_750del |
|  | *EGFR* | T790M |
|  | *FGFR3* | CNV |
|  | *FLT4* | CNV |
|  | *HRAS* | CNV |
|  | *LMO1* | CNV |
|  | *PKD1* | CNV |
|  | *RB1* | CNV |
|  | *SOX2* | CNV |
|  | *TP53* | R249W |
| 153 | *EGFR* | L858R |
|  | *EGFR* | T790M |
|  | *TP53* | A994-2T |
| 154 | *EGFR* | 746_751del |
|  | *TP53* | P278T |
| 156 | *BRAF* | V600E |
|  | *EGFR* | 746_751del |
|  | *TP53* | R248Q |
|  | *CCNE1* | CNV |
|  | *TGFBR2* | E125fs |
| 157 | *EGFR* | L861Q |
|  | *EGFR* | R776C |
|  | *KDR* | CNV |
|  | *KIT* | CNV |
|  | *PDGFRA* | CNV |
|  | *APC* | S1081fs |
|  | *SMAD4* | F35fs |
| 158 | *ALK* | HIP1-ALK |
| 161 | *MAP2K1* | E203K |
| 162 | *EGFR* | 746_750del |
|  | *EGFR* | CNV |
|  | *MET* | CNV |
|  | *TNFRSF14* | C165fs |
|  | *TP53* | R273H |
| 170 | *EGFR* | L858R |
|  | *EGFR* | T790M |
|  | *TP53* | P177T |
|  | *TET2* | H1904R |
| 171 | *EGFR* | E709K |
|  | *EGFR* | G719A |
|  | *PIK3CA* | N345K |
